# Addressing the challenge of high-priced prescription drugs in the era of precision medicine: A systematic review of drug life cycles, therapeutic drug markets and regulatory frameworks

**DOI:** 10.1371/journal.pone.0182613

**Published:** 2017-08-16

**Authors:** Toon van der Gronde, Carin A. Uyl-de Groot, Toine Pieters

**Affiliations:** 1 Department of Pharmaceutical Sciences, Utrecht Institute for Pharmaceutical Sciences (UIPS), Utrecht University, Utrecht, the Netherlands; 2 Institute for Medical Technology Assessment, Department of Health Policy & Management, Erasmus University, Rotterdam, the Netherlands; Deakin University, AUSTRALIA

## Abstract

**Context:**

Recent public outcry has highlighted the rising cost of prescription drugs worldwide, which in several disease areas outpaces other health care expenditures and results in a suboptimal global availability of essential medicines.

**Method:**

A systematic review of Pubmed, the Financial Times, the New York Times, the Wall Street Journal and the Guardian was performed to identify articles related to the pricing of medicines.

**Findings:**

Changes in drug life cycles have dramatically affected patent medicine markets, which have long been considered a self-evident and self-sustainable source of income for highly profitable drug companies. Market failure in combination with high merger and acquisition activity in the sector have allowed price increases for even off-patent drugs. With market interventions and the introduction of QALY measures in health care, governments have tried to influence drug prices, but often encounter unintended consequences. Patent reform legislation, reference pricing, outcome-based pricing and incentivizing physicians and pharmacists to prescribe low-cost drugs are among the most promising short-term policy options. Due to the lack of systematic research on the effectiveness of policy measures, an increasing number of ad hoc decisions have been made with counterproductive effects on the availability of essential drugs. Future challenges demand new policies, for which recommendations are offered.

**Conclusion:**

A fertile ground for high-priced drugs has been created by changes in drug life-cycle dynamics, the unintended effects of patent legislation, government policy measures and orphan drug programs. There is an urgent need for regulatory reform to curtail prices and safeguard equitable access to innovative medicines.

## 1. Introduction

Global health care expenditures have been rising sharply, and drug costs are a major factor.[1] Recent public outcry about exceptionally high prescription drug prices have made this subject a popular media and political topic. Discussion of drug prices has moved from an academic and government level to a broader society level and now includes the evaluation of public impact. The price of medicines was one of the campaign issues in the 2016 Presidential election in the US [2;3].

Many examples of high drug prices exist and are frequently discussed in the media. They include several types of therapeutic drugs and geographies. One often mentioned example is imatinib (brand name Gleevec^®^), a drug for chronic myeloid leukemia,which tripled in cost after the US Federal Drug Administration (FDA) allowed for a new indication. Novartis raised its price from $31,930 in 2005 to $118,000 per year in 2015 despite a huge increase in the volumes sold. The price hike occurred despite the fact that research costs for the new indication were included in the initial price.[4–6] Also in the US, the list price of sofosbuvir (Sovaldi^®^) is $84,000 for a 12-week treatment, or $1,000 a pill,[7] which has caused health plans to refuse routine coverage of this drug for hepatitis C virus (HCV) infection.[5;8] Sovaldi^®^ alone accounted for 64% of US HCV-related spending in 2014, which totaled $12.3 billion.[9] Sovaldi^®^ could be cost effective, since it prevents the ultimate need for a liver transplant, but the financial impact is too high for US insurance companies to make it available for all patients with HCV infections.[2;10;11] In Spain and Latvia, the cost of a complete treatment of Solvadi^®^ was noted to be “unsustainable” by key stakeholders such as pharmacists and pharmaceutical policy experts.[12] The cost of an alternative combination of ledipasvir/sofosbuvir (Harvoni^®^), marketed by the same pharmaceutical company, Gilead Sciences, is comparable to a course of Sovaldi^®^.[13] But the high prices for HCV drugs are not the exception. In another example, US patients suffering from cystic fibrosis were denied reimbursement for a new drug–ivacaftor (Kalydeco^®^), with an annual cost of $311,000[13;14]–unless their health worsened on older, cheaper treatments.[13] The cost of pyrimethamine (Daraprim^®^), a 60-year old drug, rose from $13.50 to $750 per pill (a 5455% raise) after Turing Pharmaceuticals acquired the distribution licence. This has further sparked public debate.[15–17]. Additional price hikes in Mylan’s EpiPen^®^ from $94 ten years ago to $609 for a pack of two have caused additional public backlash, protests and US Congressional hearings.[18–20] The results of a recent trial,[21] which show that 74 patients needed to be treated for two years with the new cholesterol-lowering Evolocumab (Repatha^®^) to prevent one cardiovascular event, indicate that with the current list price of $14,523 per year, the prevention of one event would cost over $2 milion.[22] These and many more examples of high prices for medicines, however innovative, are untenable and frequently beyond the ability of individuals, health insurance companies or even governments in high-income countries to pay[23].

Governments and health insurers are struggling with the dramatic increase in costs of new medications.[7;24–26] In December 2015, the US Senate issued a warning report on Sovaldi’s escalating drug price and its impact on the US health care system. The committee report said the Gilead Sciences pharmaceutical company had set the price as a benchmark to “raise the price floor” for its future hepatitis C-drugs like Harvoni, thus knowingly reducing the number of eligible patients for these superior treatments to cure HCV.[9] US congressional committees have opened enquiries into similar drug-pricing practices.[27] Simultaneously, on the other side of the Atlantic, the UK cost gatekeeper, the National Institute for Health and Care Excellence (NICE), initially rejected reimbursement for two costly cancer immunotherapies—nivolumab (Opdivo^®^) and trastuzumab/emtansine (Kadcyla^®^)—despite fierce opposition by industry and patient groups.[28] In both cases, the costs were estimated to amount to £90,000 per patient per year.[29;30] With a number of better targeted immunotherapies–that fit within highly promising precision medicine approaches–on their way to the market, the drug pricing and funding crisis is expected to deepen and reach a critical level for even the wealthiest countries.[31] The German government is planning to curb companies’ right to set launch-prices. Belgium, Luxembourg and the Netherlands are working together to seek a common approach to their price negotiations with drug firms. A January 2017 Lancet commentary co-authored by the Dutch Minister of Health Edith Schippers stated that: “We need meaningful efforts by both the pharmaceutical industry and governments to invest in new medicines, provide full transparency on costs, prices, and who pays what beforehand, and respect the legal space for governments to protect public health. If we don’t succeed in these efforts, we cannot guarantee people’s access to innovative and affordable medicines”[32].

On average, countries in the Organization for Economic Co-operation and Development (OECD) spend 17% of their health care budgets on pharmaceuticals;[24;33] in some countries, this is even more.[25;34] For low- and middle-income countries (LMIC), drug expenditure can be a critical public health problem[35–38] with some drugs out of reach for even well-insured patients.[26;39] In some cases, to prevent striking increases in premiums or taxes, regulators are forced to limit access to healthcare,[13;24;40–42] which leaves patients without the best treatments.[43] Of concern, is that the pharmaceutical industry might be tempted to view these high-priced models as the direction for future drug pricing of new drugs that impact larger populations[13;44–46].

The prescription drug price controversy is not new. In the 1990s, there were comparable heated debates on the high prices for interferons, paclitaxel (Taxol^®^) and HIV/AIDS medication.[1;47;48] Though the prices of these drugs were much lower than current new drug price levels, the fact that taxpayers had helped to pay for developing those innovative therapies at the time, generated public debate on fair pricing. In LMIC, where the need for HIV/AIDS medication was the highest, the fair-pricing issue was even more pressing, particularly with regard to the problematic availability of essential HIV medicines[38;41].

Pharmaceutical expenditures are based on two factors: price and volume. This means that regulation can either aim to lower drug prices, or reduce usage.[34;49;50] On the one hand, there is a growing life expectancy (and aging population worldwide), while there are increasing medical options for disease control.[51;52] Therefore, following drug innovation expectations and usage growth statistics, it is likely that costs will continue to rise.[53;54] Many countries are striving towards universal health coverage, with guidance from the global public community,[55–57] to reduce individual catastrophic spending.[58] Although these countries are preventing individual catastrophic spending by pooling risks and costs, a sustainable solution to the problem of fast-rising drug costs is still necessary. The solution will require unprecedented measures to prevent health care costs from spiraling out of control[59].

Many articles have been written about the high cost of drugs. Most seek to define the cause of high drug prices in terms of government policies or industrial pricing strategies and propose related policy measures to combat the phenomenon. This review takes another angle, and presents a comprehensive analysis of the long-term dynamics of pharmaceutical markets, drug life cycles and the sometimes unintended, counterproductive effects of market interventions by governments and health insurers. The aim is to determine what has caused the recent exponential rise in drug prices, and what can be done in terms of measures around drug pricing to safeguard equitable access to innovative medicines.

The article is structured as follows. First, drug life-cycle dynamics are discussed. Next, government interventions aimed at reducing drug prices and their consequences are highlighted. Finally, we provide suggestions for alternative policy measures to reduce drug prices and improve access to innovative and essential medicines.

## 2. Method

The prescription drug price controversy has been developing for several years now, but 2015 brought a significant change in public perceptions. Several new expensive drugs were introduced, and even old drugs were subject to price hikes. To find causes and possible solutions for recent price increases, a systematic review was performed.

Due to the wide selection of journals, PubMed was used as the search engine for peer-reviewed scientific articles. The PubMed search was performed on February 24, 2017. The search strategy was (Drugs OR medicines) AND (prices OR costs) NOT (Cost-effectiveness OR Clinical Trial OR treatment), published between January 1, 2014 and January 1, 2017, in English, and with full text available in PubMed. The articles were screened by TvdG based on the title and abstract to determine inclusion, and then read in full. Additional scientific articles were included based on the reference lists of selected journals since due to an inherent time-delay, systematically searching for topic-related articles in scientific peer-reviewed journals provided insufficient coverage of the controversy.

To include the most recent developments, additional searches in the databases of a select number of reputable English-edition international financial and daily online newspapers were performed. For this purpose, the Financial Times (FT), the New York Times (NYT), the Guardian and the Wall Street Journal (WSJ) were selected to ensure insight from both an American and European perspective, and from both financially focused and general newspapers.

**Fig 1:**
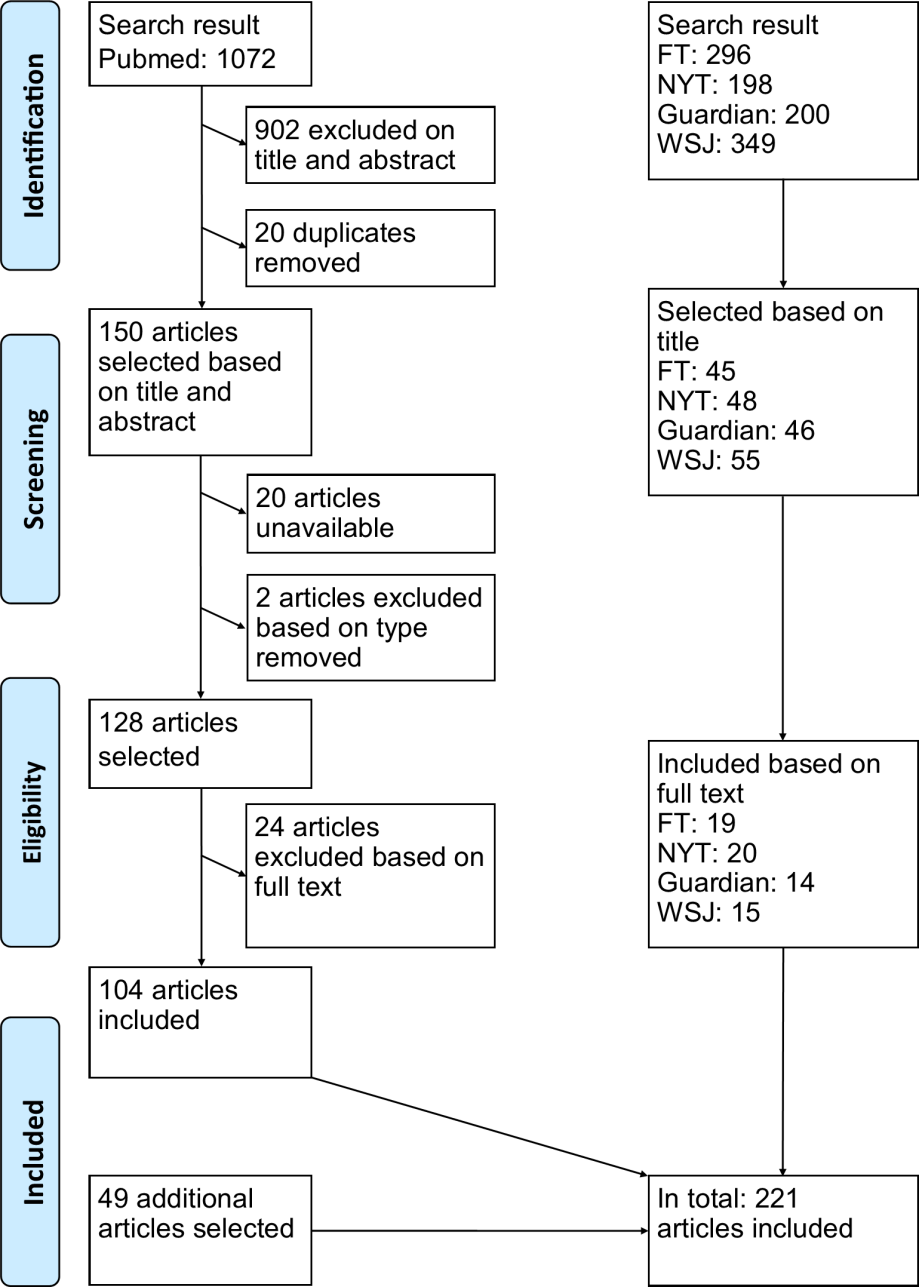
PRISMA flow chart. Schematic overview of the study selection process. FT: Financial Times, NYT: New York Times, WSJ: Wall Street Journal

The newspaper articles were limited to those published between January 1, 2015 and December 31, 2016. For the FT and the NYT the terms “Drug AND Pricing” were used in the LexisNexis search engine due to the availability of articles. The Guardian was searched for the exact combination “drug prices” using the Google-based search engine on their website. The WSJ was searched with ProQuest, with “Prescription AND Drug AND Prices” as search terms. The news articles selected by title only were all read and selectively added until there was a saturation of citations and information. The result of the searches and selection procedure is displayed in Fig 1. To ensure the quality of reporting, the Prisma checklist was used.[60] This study was not registered with PROSPERO.

Inclusion criteria

- Published in peer-reviewed journal or selected newspaper
- Published between January 1, 2014 and December 31, 2016 for scientific articles
- Published between January 1, 2015 and December 31, 2016 for newspaper articles
- Published in English

## 3. Life cycles and market dynamics

The justification for high prices for pharmaceuticals can be seen as part of the changing nature of drug life cycles and market dynamics. Further details on both these aspects are presented in this section.

**Fig 2:**
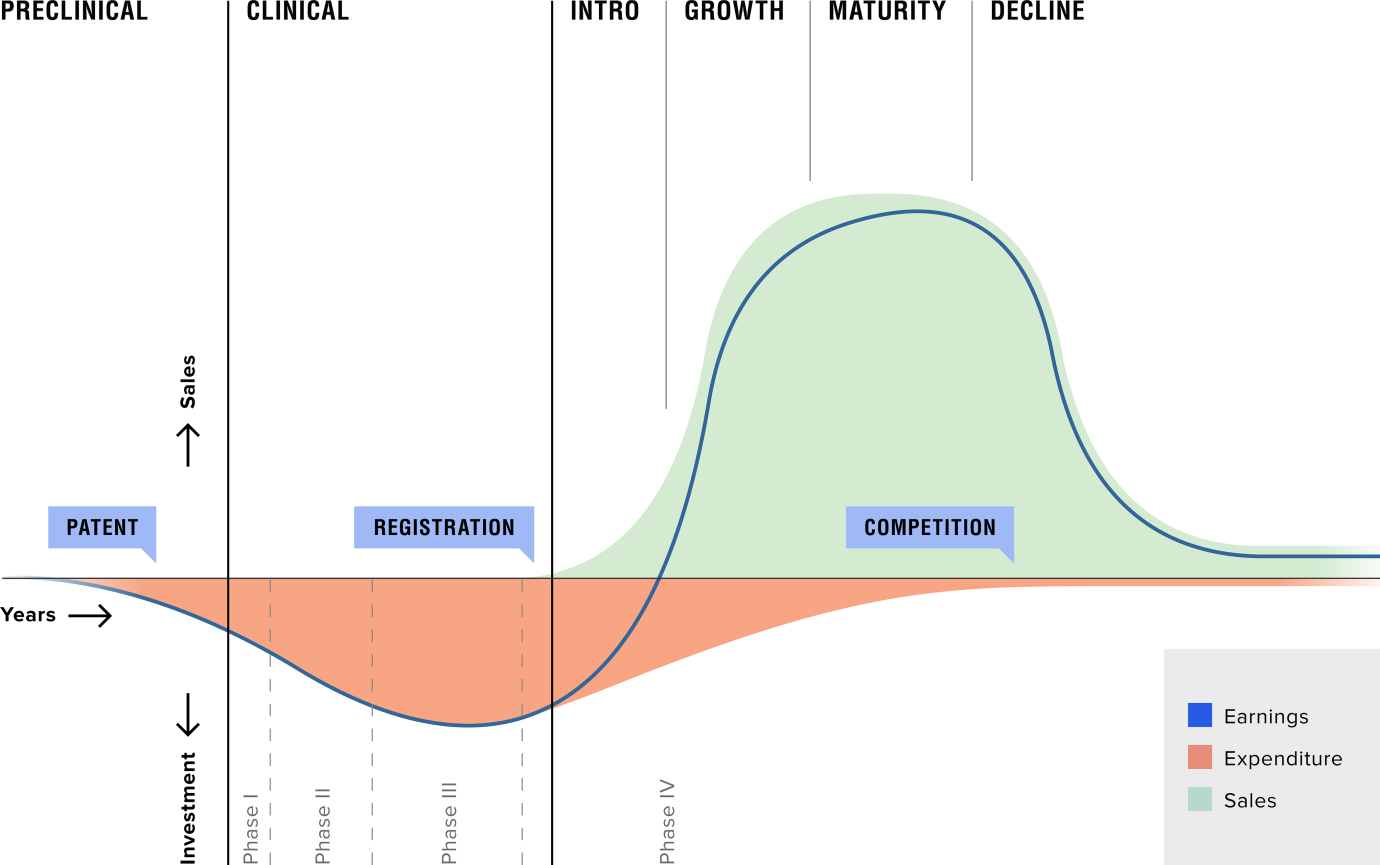
Drug life cycle curve. General curve describing an innovator drug’s investments and earnings during R&D and market performance. The life cycle phases are indicated above the graph, and the phases of the R&D trajectory are below the graph. Own work.

Life cycles describe the market behavior of many products. Generally, the product life-cycle pattern is represented by a “bell shaped” graph, a parabola, as exhibited in Fig 2. Though specifics can vary wildly, the general shape of the curve of investments during the drug development phase, exponential growth of sales after registration and decline through competition and patent term expiration is valid for most drugs.[61] Drug life cycles generally have four stages. First, there is a testing and approval trajectory. Second, after the drug is introduced there is market expansion, and the product is accompanied by growing expectations and drug indication extension. Next, drug maturity with a high sales volume is accompanied by rising criticism and disappointment regarding drug effectiveness and side-effects. Finally, there is contracting use and limited drug application. In most cases, this is a gradual process that involves the documentation of less favorable experiences and reports of the drug’s effectiveness and adverse reactions in everyday practice. Thus, a drug’s benefit-risk assessment and the resulting safety profile is under constant revision. Over time, newer and presumably better alternatives gain attention. This is part of an evolutionary process of selection and adaptation. Most brand-name medicines continue their careers as generics after their patents expire. On average this results in a 20–25 year therapeutic life-time in ‘the doctor’s bag’–the portfolio of drugs available to a doctor–due to therapeutic substitution and competition between branded drugs and generics[62].

What we tend to forget is that therapeutic drugs are inextricably linked to a dynamic mixture of opinions, practices and rituals and as such are important tokens of healing as a cultural process. Doctors, regulators, health care insurers, patients and politicians, however, prefer to believe that it is evidence-based medicine and not enthusiasm or belief about a particular drug that makes a drug more or less therapeutically effective. Dan Ariely, a behavioral economist at the Massachusetts Institute of Technology, and his research team have shown that a 10-cent pill doesn't kill pain as well as a $2.50 pill, even when they are identical placebos.[63] This may partly explain doctors’ and patients’ preferences for high-cost brand-named drugs over inexpensive, widely available, chemical and therapeutic generic equivalents[64].

### 3.1. Types of prescription drugs

This article refers to prescription drug prices, but there are distinct types of prescription drugs and this requires clarification. First, there are drugs that are under patent, with an exclusive producer and no direct competition. Then, there are generic drugs with an expired patent that allows for production by other manufacturers.

Biological drugs follow the life-cycle patterns of small molecules or conventional drugs, but higher prices are accepted and specific regulation of generic competition is in place. Oncological drugs are a separate category, because high prices are historically more common,[26] expected to rise,[5] and more acceptable given the severity of the indications. Laws are in place to incentivize the development and marketing of orphan drugs, which means they follow market dynamics that differ from conventional drugs. Finally, when the patent runs out, and other producers can manufacture the same drug, generics are introduced. In the case of biologicals, biosimilars compete with the innovator while following a specific set of regulations[65].

### 3.2. Patents and registration

The pharmaceutical industry is often characterized as a competitive sector in a free market, where the total supply and demand determine market price. However, according to business analysts, in a truly free and competitive market without patent regulation, it would be difficult to profit from new drug development.[66] This is why governments protect companies from competition during the life of a patent. In general, the term of a new patent is 20 years from the date on which the application for the patent was filed.[1;40;67;68] This can be extended to 25 years.[69] In addition, in the US, the FDA can grant exclusive marketing rights upon a drug’s approval, which is generally concurrent with the length of a patent. The FDA usually grants new drug exclusivity for between seven years for orphan drugs[70] and five years for new chemicals, with an additional period of six months of exclusivity following pediatric approval[71;72].

Patents are also granted for new chemical entities. This allows companies to charge high prices once the drug is ready for marketing.[1;73] Patents then become public, which gives other producers the chance to further improve and develop the drug[41].

Patent timelines are limited, which provides an incentive for companies to shorten the drug development phase or look for disease areas with less stringent trial requirements. For example, there is more research in drugs for late-stage cancer than early-stage cancer, because of the less demanding and shorter trial trajectories[1;74;75].

The number of patents a company files, or alternatively the research and development (R&D) costs per patent filed, are often used as an output measure for the efficiency of drug development and the future of a firm.[76] Since most patented molecules do not make it to the market as an actual medicine, both datasets are incomplete representations of productivity[77].

In debating the patent system, some analysts state that basic human rights like health and access to essential medicines should be equitable[23] and should not be limited by property rights.[1;6;78;79] Others use a utilitarian stance to argue that pharmaceutical companies are for-profit entities,[40;73] and without patents these companies would not be incentivized to develop drugs.[1] This difference in viewpoint is illustrated by the litigation surrounding patents and compulsory licensing (see paragraph 4.2.7) in LMIC[73;80;81].

### 3.3. Developmental phase and registration

Pharmaceutical companies must register new drugs, which requires clinical studies and safety tests. This is a high-risk, high-cost and low-output endeavor. The odds of having a drug approved varies from approximately 24% (for systemic anti-infective drugs) to less than 10% (for drugs used to treat cardiovascular, gastrointestinal or metabolic disorders).[82] On average, it takes a company ten years to register a drug.[41;68;77] Thus, companies have to decide on projects that have a good chance of becoming registered drugs several years in the future.[83] The drug development process requires investments, estimated at between $60 million to $2.6 billion,[6;67;68;77] though most estimations are close to $800 million from bench research to prescription medicine.[33;66;72;84;85] The wide range of cost estimates is due to the lack of clear data and various methods of calculation, and depends on the type of drug and the trial data required,[72] as well as the size of the company developing the drug.[86] Development costs are highest for large companies due to their relatively high overhead and marketing costs[1;76].

Historic examples illustrate what happens when the demonstration of medicine safety during development is not adequately regulated. An exemplary case is the thalidomide drug disaster that took place between 1958 and 1962.[24;71;74] This drug for morning sickness resulted in malformations in the extremities (phocomelia syndrome) of thousands of babies born to women who had taken thalidomide during pregnancy.[71] Regulatory reaction to drug safety alerts often involves the introduction of more stringent regulations requiring more safety and efficacy studies, which leads to more dropouts in the development process and an increase in invested time and costs.[41;74] Regulatory agencies are criticized by many parties for being either too stringent (delaying innovation and increasing costs) or not stringent enough (allowing dangerous drugs to be marketed).[72] Arthur Daemmrich, a US historian, discussed this tension between safety management and drug innovation and was the first to use the term ‘double bind trade-off phenomenon’[87].

The imperative of regulation makes it more difficult for smaller companies to register drugs, thus limiting the number of firms with the critical mass and financial means to invest in drug research. This situation limits viable competition from smaller companies[41] for Big Pharma—the collective sector of large pharmaceutical companies. That is why most new drugs that received a positive reaction from the Committee for Medicinal Products for Human Use (CHMP) of the European Medicines Agency (EMA) between 2010 and 2012 were filed by large (59%) or intermediate-sized (28%) companies.[88] Small enterprises are important during early phases of development. However, in later phases, if the success of a new chemical entity developed by a small company is likely, a large pharma company will often buy the small company or purchase the licence for the new medicine[88].

R&D for medicines has been declining.[41;67;68;89] Higher investments, however, will not necessarily fill R&D pipelines with new promising drug compounds. R&D has recently yielded fewer drugs than in years past, since low-hanging fruits have already been harvested.[66;89;90] Furthermore, there are many drugs with promising results in phase II settings that have not made it to phase III settings.

Regulatory agencies allow drugs to be released to the market based on safety and effectivity, but not with reference to price or cost-effectiveness.[70;91] This means the price and reimbursement of a drug are determined only after registration approval and insurance company and/or government negotiation[91].

### 3.4. Post registration and reimbursement

Once a drug is registered for a specific disease indication, manufacturers can apply for reimbursement. Many public health care systems allow the government to control drug prices. Some base the acceptability of a price on the Incremental Cost Effectiveness Ratio (ICER) and budget impact.[10;83;92] This means companies have to assess the volume of sales[93] and the price at which they are reimbursed, and then offer a price based on that estimate.[90;94] Then, negotiations take place between the company and the reimbursing agent or government to determine an acceptable price for each stakeholder.

A drug’s reimbursed price can be lower than the pharmacy retail price or list price. This makes patients aware of drug prices, since they will have to pay for the difference out of pocket. Such pricing and reimbursement schemes can be a tool to make patients switch to cheaper or generic drugs, and make manufacturers of high-priced drugs lower their prices to prevent patients from making this switch.[36;59;95] Manufacturers argue that patient co-payments can cause adherence problems,[96;97] especially for expensive[6;98] and psychiatric drugs.[99] This means physicians and patients prefer drugs without co-payments.[78;100] To circumvent this situation, producers have implemented patient-assistance programs, which are discussed in paragraph 5.6.

Companies want to make the highest possible profits in each country by differentiating prices,[40] but they also want their prices to be similar across countries and close to competitors to reduce the incentive for parallel importation.[24;101;102] Governments worldwide want innovative new drugs to be available as quickly as possible, so their population can profit from them. High drug prices may incentivize companies to develop and launch their new drugs faster.[1;40;73] On the other hand governments also want to have affordable drugs for everyone at the lowest possible price, to reduce healthcare spending[1;5;78].

In the US, the government does not control reimbursement,[16;33;91] based on the assumption that the free market will drive the pharmaceutical industry to compete, which will result in lower prices.[92;103–105] Thus, pharmaceutical companies set their own prices, which allows for market calculations aimed at maximizing profits.[106–108] As a consequence, US prescription drug prices are among the highest in the world.[6;33;109] For the uninsured, a cancer diagnosis is still a major cause of personal bankruptcy[110;111].

In general, the manner in which drug list prices are determined is not transparent,[14;57;106;112] so critics are pushing for more transparency.[77;98;113] Some differences in pricing between countries can be explained by differences in health care systems[4;93;114] and socio-economic dynamics,[78;84] all of which lead to differences in ICERs. In some cases, a drug’s price is based on the old standard of care plus a premium,[5;6] the uncertainty associated with the drug[93] or simply the government’s willingness to pay.[92;115] Ultimately, a drug’s price is unrelated to the cost of development[4;5;51] or a country’s gross domestic product,[12] and only related to cost-effectiveness if the payer introduces this into negotiations[30;53;107].

### 3.5. Introduction and growth phase

**Fig 3:**
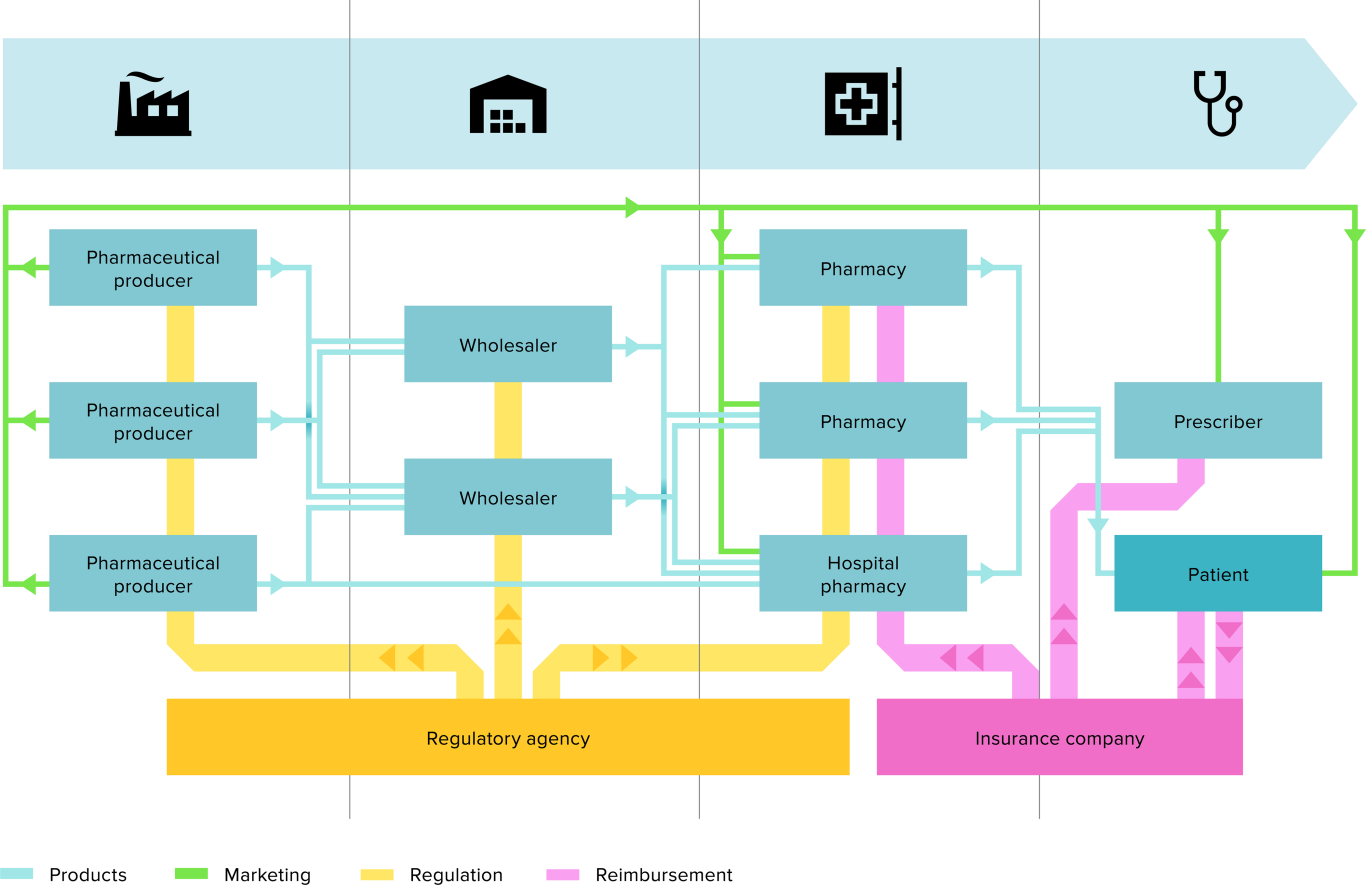
Stakeholders. A simplified, schematic overview of stakeholders and relationships in the pharmaceutical market. Own work.

When a drug is marketed, there is a level of market penetration.[61] It is important to the producer that the drug is brought into use as quickly as possible, since a patent limits the period of exclusivity and thus profit. Speeding up market penetration was historically accomplished by advertising to doctors, pharmacists and patients (see Fig 3).[116] In the US, doctors are paid directly for promotional activities,[112] though the Affordable Care Act (ACA) requires disclosure.

Direct-to-consumer advertising (DTCA) is controversial, since it increases demand, partially through inappropriate prescription. Doctors will prescribe drugs that are not indicated if they know they can satisfy their patients by doing so, or unnecessarily prescribe branded drugs. This increases costs without improving health.[117] Therefore, this practice has become illegal in most countries, but not in the US or New Zealand.[1;117–199] This situation makes it harder for drug companies to create demand for their products in most countries, and reduces turnover in the early phase of developing a new drug.

In Europe, marketing to doctors and pharmacists is permitted, if it is medically substantiated.[120] This requires more expensive studies, and careful wording of the marketing message. Still, marketing is a large part of the pharmaceutical industry’s expenses. In fact, more money is spent on marketing than on R&D[24;41;121].

To market drugs to doctors and circumvent this regulation, trials are sometimes used as a marketing tool.[48] ‘Seeding trials’ are designed to ‘seed’ the use of a drug among patients and physicians, while they often offer no scientific purpose.[122;123] Furthermore, medical centres that want to promote an image of being at the cutting edge of science are willing to test the newest available experimental treatments and compete for hosting profitable industry-sponsored clinical trials.[48] This was the case for interferons when companies were looking for an indication for this new experimental drug.[48] Numerous promising new drug compounds have followed suit[122;123].

A pharmaceutical company’s marketing push is somewhat mitigated by the increased tendency to work with local guidelines.[25;50;73;124] This often improves the quality of care for the patients, but makes it harder for new drugs to enter the doctor’s bag. In addition to the cycle of updating those guidelines–which generally happens once every five years, or less frequently[46]–new drugs have to show superiority to already available drugs. Furthermore, the price of a new drug is often higher than that of older drugs that might be available as generics, which increases the demand for proven superiority[41].

### 3.6. Mature phase

During a drug’s patent life, doctors and pharmacists play a crucial role in the choice for one drug over another.[1] These professionals need to inform the patient about their pharmaceutical options, and a drug’s effectiveness and costs. This is why advertising aimed at brand recognition continues during the mature phase of a drug’s life cycle[61].

Several studies have shown that if there are financial incentives for doctors to choose one drug over another, the one that is most beneficial to the doctor’s finances is most likely to be prescribed.[1;125] In the US, where Medicare and Medicaid reimbursement is based on a 6% mark-up of the price of cancer drugs,[53;110;119] doctors have an incentive to select the more expensive option.[1;5] This is another explanation for the high prices for drugs in the US. In order to help patient and doctors, the European Society for Medical Oncology and the American Society of Clinical Oncology have developed frameworks to assess the value of new cancer drugs[58;126].

To combat incentives to prescribe expensive drugs, some health insurance companies in the US offer monthly payments of $350 to physicians who prescribe according to their guidelines, thus saving costs and improving patient health.[5] This scheme compensates a physician for earnings missed by prescribing less expensive drugs[5].

In this phase, companies often attempt to have their drug registered for additional indications, thus increasing the number of patients, to increase their sales volume. A larger patient base would logically make the cost per treatment lower, but this is often not the case[53;111].

### 3.7. Declining phase

Several countries around the world have implemented preference policies, aimed at generic substitution.[1;127;128] This policy requires physicians to prescribe and pharmacists to dispense the cheapest available version of a drug, often generic, unless a more expensive one is medically necessary. This can be the case for drugs with a very small therapeutic window, like Tegretol^®^ (containing generically available carbamazepine) for the treatment of epileptic seizures.[127] For these drugs, the preference policy implies that new patients start on a generic drug, but those who have already reacted well to a branded version do not have to switch. The potential substitution rate differs per indication group[103;127;129;130].

Generic substitution reduces the length of the life cycle of patented innovator drugs, since they are replaced by generics as soon as the patent expires. The time an innovator drug has to generate income is thus reduced, which makes higher prices during the exclusive phase necessary to generate the same turnover.

Doctors and patients are often hesitant to change the brand of the drug after patent expiry, since they are familiar with the innovator and trust it.[69;96;99;128;131–133] This explains why generic substitution only happens on a large scale if policy to enforce it is in place. In some countries, generic substitution by pharmacist’s initiative is mandatory;[78;130] in others it is prohibited[99;130].

Since roughly 2000, Big Pharma has been struggling with the patent cliff, a series of blockbuster drugs whose patents have expired. This has caused a significant loss of turnover due to generic substitution.[66;67] The effect cannot be compensated for by new drug introductions, since relatively few new blockbuster drugs have been introduced.[103] This means that in order to maintain profitability, more revenue must be generated from fewer breakthrough drugs, which has led to increased prices for innovator drugs and increased merger and acquisition activity within the pharma industry. Over the last two decades, 60 pharma companies have merged into just ten pharma companies. This consolidation has helped Big Pharma gain more power to influence regulation and pricing policies while simultaneously diminishing competition (see Fig 3)[134].

The patent expiry and substitution effect is expected to be smaller for the new generation of biologicals, because these biological drugs are more difficult to copy. Also, a drop in price in cases of biosimilar substitution is often just 20–30%,[65;85;135] whereas the price for conventional small molecules can fall by more than 70%[85;99;131].

Large pharmaceutical companies employ several tactics to extend the life cycles of their products, and reduce their loss of income due to patent expiry. Strategies to address this often include improved formulations (e.g. Seroquel^®^ XR, quetiapine), new indications (e.g. Zyban^®^, bupropion), chiral switching (e.g. omeprazole (Prilosec^®^)/esomeprazole (Nexium^®^) or citalopram (Celexa^®^)/escitalopram (Lexapro^®^)), combining drugs (e.g. Harvoni^®^, containing ledipasvir and sofosbuvir), changing to over-the-counter (e.g. Prilosec^®^, omeprazole) and finally introducing an authorized generic[6] (e.g. atorvastatin, Lipitor^®^/Zarator^®^)[67].

Another option is the development of pediatric dosage recommendations and formulations, which allows for extension of market exclusivity by the FDA for six months.[71;72] Finally, ‘pay for delay’, the act of paying off generic competition so an innovator can maintain market exclusivity, is fiercely contested by both the European Commission and US Federal Trade Commission, but the practice has been increasing over the last few years[67].

Some drug brands are so strong that, even after the loss of market exclusivity, doctors and patients continue to privilege them over generic drugs. Examples include brands like Viagra^®^, Prozac^®^ and Aspirin^®^.[136] For over-the-counter medicines, in particular, branding is a relevant mechanism to maintain market share, since consumer name recognition is a more important factor in product choice when there is no medical professional role.

Fierce competition between generics can cause companies to offer prices that become too low to be sustained by the offering company. Sustainability can be threatened by the rising prices of raw materials and production costs,[137;138] which lead to a failure of supply and shortages.[95;131;138] This situation can also force competitors for a specific drug to completely retreat from the market, which leads to fewer producers[139] or even a new monopolistic position for a generic,[137;140] as recently seen with Lanoxin^®^ (digoxin) and Daraprim^®^ (pyrimethamine). The result is that even for generic medicines we increasingly see steep price increases.[141].

### 3.8. Changing life cycle dynamics, effects on drug pricing and profitability

Drug life cycle analysis indicates a trend of shortening life cycles and pharmaceutical companies experiencing more difficulty achieving high, sustainable sale volumes during the past two decades than before. Since a company’s income is based on volumes multiplied by price (equals value), the first strategy to maintain high revenues is to increase price.[142] Despite regulated pricing, this practice results in drug spending growth matching overall medical spending growth[39].

On average, the top ten pharmaceutical companies have a profit margin of 20%;[2] those noted in the S&P 1500 have a net profit margin of 16%, compared to 7% for all other companies in the index.[119] This means that even though companies experience more difficulty in achieving long-term high-volume prescription drug sales, the higher drug prices compensate for the lower product turnover and safeguard Big Pharma’s high-profit profile. This is not surprising, because pharmaceutical companies are for-profit entities that wish to maximize their profits and increase share-holder value without breaking the law.[24;40;143] However, this approach means they may not automatically do what is best for society. Critics argue that more regulation is needed to counterbalance Big Pharma’s only-for-profit motive and force them to do what is best for all stakeholders.[15;53;104;144;145] Through a number of interventions (some more effective than others) governments and their regulators have tried to direct either the price of drugs or the availability of innovations. Government interventions to stimulate or curtail the pharmaceutical markets and the introduction of new procedural measures concerning drug patent licences and drug registration licences are discussed in the next chapter.

## 4. Drug innovation, regulation and pricing interventions

As stated previously, though the pharmaceutical market is often portrayed as a competitive market, it is not truly a free market. In addition to the patent system, skewed economic dynamics create further complexities. In free markets, a consumer decides on, buys, pays for and uses a product, whereas in healthcare, a doctor decides and the pharmacy or hospital pharmacy provides, the insurance company or government pays and the patient uses the product.[142;146] An overview of the stakeholders and their relationships is given in Fig 3. Financial incentives are not aligned with consumption, so companies’ pricing power is not related to how consumers value the products.

### 4.1 Unintended consequences of innovation and measures to stimulate drug safety

Drug regulation usually aims at making new drugs available while keeping costs down, however, there can be unintended consequences. For example, if drug production or distribution becomes too competitive to remain lucrative, the market can become so consolidated that drugs are no longer available.[140] Even worse, temporary or sustained monopolistic positions can arise due to market failure which can cause an increase in prices.[104;106;147] Hence, after specifically designing a policy to stimulate innovation and safety or reducing prices, potential consequences should be carefully monitored.

#### 4.1.1 Orphan and priority drug regulations and potential consequences

There are cost-reduction strategies that could work by changing the trajectory of a drug’s development.[53;68] The first option is to speed up innovation and regulatory approval, so that companies have less waiting time before marketing a drug and thus enjoy a longer profit-generating post-marketing patent life.[5] One way to do this is to accept surrogate parameters as trial endpoints to prove efficacy, which saves time.[135] Another innovative option is to harmonize regulation between countries, so companies only have to prove efficacy once.[135] However, because drugs are not necessarily priced based on investment costs, the effects of this approach on drug pricing might not be significant, or as we will see, harmonized regulations may actually be counterproductive.

One clear example where regulation is in place to speed up innovation is the field of orphan drugs. Generally, low patient volumes make it unattractive for pharmaceutical companies to invest in the development of orphan drugs.[148–150] Fabry disease, for example, has a prevalence of approximately 1 per 100,000 persons, thus making it unattractive for companies to develop drugs for these patients without further incentives.[149] That is why the orphan drug regulation was designed.

The benefits of developing drugs with an orphan status are exclusive licensing for seven years, faster assessments and lower taxes in the US. European regulatory bodies offer exclusive licensing for ten years, lower regulatory fees and scientific advice.[148;150] Orphan drug legislation has worked, yielding at least 73 drugs for orphan indications in the European Union since the law passed in 2000[151] and 335 in the US since the FDA set regulations in 1983 and 2002[152].

Another incentive for companies in the US that develop orphan drugs is a priority review voucher, which is also available for tropical disease drugs. This voucher is awarded after a company develops a drug for an orphan disease and releases the patent. When applied, it allows companies to request an expedited review process for a new drug, which can speed up the regulatory process by several months. This approach allows a pharmaceutical company to stretch the patent period, and thus the mature, beneficial part of the life cycle of a subsequent blockbuster drug.[153] A well-used voucher could increase a company’s income by up to $300 million according to some estimates.[1;154] The company can also sell the voucher to another company that is about to launch a similar drug for roughly the same price[154].

The moral argument for orphan drug regulation is that society, and medical science in particular, has an obligation to pursue new therapies for everyone, including people who suffer from orphan diseases.[149;152] The downside of this policy is the non-utilitarian outcome that money is being used for diseases that very few people have, and that the same money could have been used for more relevant research reaching a larger population[1;145;151;152].

Given that no price ceiling or maximum budget impact is imposed on an orphan drug’s regulatory design, orphan drugs are often very expensive. Prices are unrelated to effectiveness or prevalence, which means that regulatory bodies frequently label these drugs as not cost-effective.[148;151] Also, the evidence for the effectiveness of orphan drugs is often lower in quality than required for regular drugs, and more side effects are tolerated than for other drugs[148].

In Europe, market exclusivity can be withdrawn after five years if a product has generated adequate profit. However, this has not happened for a single orphan drug to date.[149;155] Even for orphan drugs that have lost exclusivity, no generic producer has ever created a competing product, and it is doubtful whether or not this would ever be attractive given the low patient numbers[151;156].

Orphan drug regulation is an example of a policy working better than expected, thereby increasing healthcare budgets. Companies have tried to abuse this regulation for profitability purposes. For example, there was a request that malaria be designated for an orphan drug indication in Europe[71].

Orphan drug regulation is also used by pharmaceutical companies to register drugs so that multiple indications–not always orphan indications–can be added. For example, Gleevec^®^ (imatinib) has been marketed for several orphan indications,[149] yielding a turnover of $4.7 billion in 2012.[6] Rituximab, the world’s second most profitable drug, also holds multiple orphan drug indications, in addition to the use for common rheumatoid arthritis.[155] Although this approach increases drug volumes, companies usually do not reduce prices.[152;157] Finally, pharmaceutical companies split common indications into groups that are small enough for their drugs to qualify for an orphan drug label[70;149;155].

Regulations for orphan drugs are quite effective, but should include compulsory price-ceiling measures to prevent tax-payers from paying twice, first for some of the R&D costs and second for reimbursement of overpriced drugs. Furthermore, the orphan drug label should only be applicable if a drug has not been granted access for another indication already. Currently, Revatio^®^ (sildenafil, also known as Viagra^®^, for erectile dysfunction) is being sold as an orphan drug for pulmonary arterial hypertension[149].

#### 4.1.2 The FDA’s unapproved drugs initiative and consequences

In June 2006, the FDA announced a new drug safety initiative with the goal of removing unapproved ‘old’ generic drugs with problematic safety profiles from the market. The FDA states that it uses a risk-based enforcement program in order to focus on products that pose the highest threat to public health and “without imposing undue burdens on consumers, or unnecessarily disrupting the market”.[158] However, the program has had unintended consequences. If a product is not officially approved by the FDA, the agency can require a New Drug Application from the manufacturer, which is reviewed to determine if the drug meets FDA standards. Inexpensive generic drugs that have been on the market for decades are studied anew, drug applications are filed and exclusive patent rights to sell the drug are given to the first manufacturer who meets the new FDA effectiveness standard. This manufacturer can then decide what to charge—with no competition. Exemplary is the patent flipping of the formerly inexpensive drug colchicine (less than $1 for 30 pills), used to treat flare-ups of gout. Now, after FDA review, just one manufacturer has the patent rights to market colchicine as Colcrys^®^, and the retail price is almost $200 for 30 pills. How drug pricing measures could counteract these pricing strategies by the pharmaceutical industry is discussed below.

### 4.2 Possible drug-pricing measures

There are many ways to reduce spending on drugs. However, all are based on one of four general intervention options[36].

1. Shift from expensive to cheap drugs, within the same class,
2. Shift costs towards patients or insurers,
3. Reduce drug prices,
4. Reduce total drug uses.

It is not clear which mechanism is the most effective, but authorities in many countries often implement policies encompassing several of the abovementioned options. An overview of possible regulations and the mechanisms through which they reduce costs are given in Table 1.

**Table 1:** Policy effects. This table lists the policies that are in effect in various parts of the world, their effects and their unintended consequences. EU: European Union, USA: United States of America, DTCA: direct-to-consumer advertising, UK: United Kingdom, LMIC: Low- and Middle-Income Countries.

Policy	Location	Mechanisms	Effects	Side effects
Patent laws	Worldwide	Increase innovation	Gives an incentive for innovation	Increased prices during patented period, reduced transparency in research
Orphan drugs	EU, USA	Increase innovation	Many new drugs have been developed	High prices, low quality evidence of effect
Biosimilars	EU, USA	Shift drugs	Availability of generic versions of biological drugs	Concerns about comparable effectivity and rare side effects due to fast market authorisation
Development cost reduction	EU, USA	Increase innovation, reduce price?	Faster entry of new drugs	Lower quality evidence
Limiting DTCA	EU	Reduce use, shift drugs	Reduced inappropriate prescriptions	Reduced awareness of new drugs for professionals and the public
Reference pricing	Several EU countries, Canada, Australia	Reduce price	Lower prices	Best payers get drugs first
Price ceilings	Several EU countries, Canada	Reduce price	Fewer price increases	Higher initial prices
Value-based pricing	Several EU countries	Reduce price, shift drugs	Decision making is more evidence-based, and treatments are rewarded for actual efficacy	Prices are set just below cost-effectiveness threshold, limitation of options
Preference policy, compulsory generic prescribing	Several EU countries	Shift drugs, shift payer, reduce price	Increased use of generic drugs	Shorter life cycle of patented drugs
Stimulate guideline adherence, pay for performance	EU, USA	Shift drugs, reduce price	Better prices and quality of healthcare	Limited options for treatment
Negotiation power through monopsony	New Zealand	Reduce price, shift payer	Reduced prices for population through increased negotiating power	Shift of costs to countries with less bargaining power
Voluntary out-licensing	LMIC	Reduce price, shift drugs	Lower prices in LMIC	Counterfeit parallel trade
Open tenders for exclusivity	Several EU countries, Russia	Reduce price, shift drugs	Reduces prices	Drug shortages due to less dynamic supply chains
Compulsory licensing	LMIC	Reduce price, shift drugs	Makes drugs affordable for LMIC	Counterfeit parallel trade
Incentivize physicians and pharmacists	Several EU countries, USA	Shift drugs, reduce price, reduce use	Directs towards prescribing cheaper drugs	Patients might lose access to more expensive brands
Profit limitation	UK	Reduce price	Lower profit margins, through lower prices or higher investments	Could incentivise companies to spend on less relevant causes

Schemes to reduce drug prices are used most often to reduce overall healthcare spending. For example, according to one calculation setting prescription drug prices 20% lower than the current list price would increase the number of users who can afford the drug by 23%, while decreasing revenues for the drug company by only 1%.[33] More examples with comparable outcomes exist.[4] Of course, the outcome is completely dependent on specific market conditions, prices and regulations. The general consensus is that reducing prices increases the number of users, and this could at least partially offset losses due to lower pricing. A popular argument against paying less for drugs is that innovation would not be financially worthwhile, and society would not enjoy the possible benefits of new innovator drugs.[77] This argument will be discussed later on.

#### 4.2.1 Biosimilar substitution regulation and resistance to substitution

Both the EMA (since 2003) and the FDA (since 2010) have regulations for accepting biosimilars to bring down the price of treatments by increasing competition without reducing safety.[65;72;159;160] However, unlike the EMA, the FDA only allows for an interchangeability label if the manufacturer has shown that a biosimilar drug has the same effect and safety as the originator or for switching between them.[65] The regulatory framework for biosimilars is designed with similar intent as regulation for small molecule generic drugs, but its effect is thought to be less significant. This is due to a smaller price difference between the originator drug and generics,[143;161] as previously mentioned. Research and production costs for designing a biosimilar are significantly higher than for designing small molecule generics.[65;135;161] Thus, innovators can prevent biosimilars market penetration by offering discounts on the original biological[135].

Furthermore, ongoing controversy on the interchangeability between biosimilars and originator drugs makes doctors and patients wary of using biosimilars.[159;162] It requires significant effort on the part of reimbursement authorities to overcome this unexpected and rather persistent unease about and resistance to the use of biosimilars, that is exploited by marketers of the biological originator companies[159].

Pharmaceutical policy is often designed based on negotiations among various stakeholders, but doctors are not always invited to the table, according to critics.[163] If the medical community were more involved in creating biosimilar regulations and substitution programs, the effectiveness of regulatory and cost-reduction policies might improve significantly[159].

#### 4.2.2 Engaging physicians and pharmacists in price reduction programs

As stated previously, physicians and pharmacists have a central role in determining which patient receives which medicine, and whether the use of expensive drugs is beneficial for specific patients.[133;163;164] Programs that provide financial incentives for prescribers to save on costs incentivize physicians to be cognizant of drug prices and have the potential to reduce pharmaceutical expenditure gradually and permanently, by either rewarding when expenses are low or enforcing penalties when expenses exceed indicative or earlier budgets.[34;49;165] After the implementation of such programs, doctors are more inclined to believe that medical costs are a relevant consideration in drug usage[166].

Just educating medical staff on drug pricing does not have a lasting effect. To change physicians’ attitudes, the prices of drugs must be considered, and constant reinforcement and easily available information is necessary. In one example, simply adding a sticker to indicate the prices of anesthetic drugs per hour in operating rooms significantly reduced their use and therefore, costs[167].

Another incentive to reduce spending is index pricing, which is similar to internal reference pricing. Drugs are classified in index groups of therapeutically interchangeable drugs. The prices for each group are determined based on the average drug used over the last period, which is frequently updated. The pharmacist is reimbursed for the price that is given for that index group, regardless of the drug that is actually dispensed. This incentivizes pharmacists to dispense the cheapest version of a drug, preferably below the index price so the difference can be kept as profit. This approach drives down the index price for the next period, thus creating a downward spiral.[36] Downsides of internal reference pricing are discussed in the next paragraph.

Finally, another way to reduce drug prices is for doctors to prescribe using the international non-proprietary names (INN) of drugs, and the brand names only when a brand is strictly necessary.[130] It is left to the pharmacist to dispense the drug based on the INN, so doctors have no financial incentives to prescribe more or specific brands of drugs[100;164].

#### 4.2.3 Reference pricing approaches

Reference pricing is a tool to set a benchmark for reimbursements.[36;95;168] Of all price control measures, this is the one with the most evidence for effectively reducing drug prices.[36;99] Reference pricing can be done in two ways: internally or externally.

Internal, therapeutic or national reference pricing is based on comparing a drug to other drugs with the same active ingredient or with comparable clinical effects within a country. The maximum price of the new drug is then based on the average or lowest price in that cluster.[1;55;99;163;168] This incentivizes companies to develop drugs for indications with no competition, particularly no generic competition, because that would bring the price down.[1;92] However, internal referencing discourages development in existing drug classes.[1] Late me-too drugs could be placed in a cheap class with therapeutic equivalents.[55;169] This method of reference pricing only works by reducing prices and shifting patients towards cheaper drugs[36].

External, or international reference pricing is used by most EU member countries.[78] It is based on comparing a new drug’s price with other countries with a comparable economic status,[36;59;170] or with differing economic status after which the outcome is adjusted to purchasing power parity.[171] The mechanism of pricing used by a specific country, such as value-based pricing, can also be a reason to refer to that country[55].

For example, Norway reviews nine countries and takes the average price of the three lowest prices.[119] These prices are then regularly updated.[99] This approach often provides a skewed picture, because it does not take into account undisclosed prescription drug rebates and discounts that most purchasers receive for prices on the official list.[78;99;126;172] This mechanism incentivizes companies to register in countries with the highest willingness to pay first.[155;170;172] Countries with a lower willingness to pay could see a drug launch delayed, or no launch at all, to prevent other countries from adjusting their prices downwards[92;99;170;173].

#### 4.2.4 Value-based pricing measures

The ideal pricing model should include the health and socio-economic benefits of a drug by deploying sophisticated out-come based compensation models.[105;174] The price of a drug should be proportionate to the added value in terms of quality of life, life years saved or tumor shrinkage.[6;24;95] This would improve the value per monetary unit spent on health care, and increase innovation in relevant areas[90;175].

Currently, cost-effectiveness analysis is used in many countries as a factor in the pricing of new drugs,[78;176] but often drugs and treatments that are not cost-effective are still reimbursed, and sometimes by specific reimbursement funds like the Cancer Drug Fund in England.[177] Vice versa, cost-effective drugs are sometimes not reimbursed at all.[178;179] A major reason for this is the lack of standardization in the practice of value-based pricing. Which factors are included and which are not varies, so value-based pricing is currently more of an art than a science.[24] Data about the effect of such schemes are contradictory.[99] One factor is that this policy has given a perverse incentive to drug companies to set high drug prices for the new generation of innovator medicines that are in line with the cost-effectiveness threshold (mostly in terms of quality-adjusted life year [QALY] and/or incremental cost-effectiveness ratio [ICER] terms) that a country is willing to pay. This also explains the differences in prices in individual countries, because cost-effectiveness thresholds differ across countries.

While not an official cut-off, the threshold for cost-effectiveness is set by the British regulators at approximately £30,000 per QALY,[155;181;182] and £50,000 for end-of-life drugs.[177;179] Other European countries use cost-effectiveness thresholds that can vary between €10,000 and €50,000 per QALY.[6] After extensive analysis, the total British health care system was found to provide care at £13,000 per QALY, which was much cheaper than many drugs.[181;182] This means that money would be spent more effectively on parts of the system other than high-priced innovator drugs.[177] Thus, the costs of high drug expenditures place a heavy burden on the health care system and may displace other high-quality healthcare services[178;181;183].

Another practical difficulty in value-based pricing is a drug’s indication. For example, Tarceva^®^ (erlotinib) is more effective for lung cancer (extends survival by 3.5 months longer than chemotherapy) than for pancreatic cancer (extended survival by two weeks versus placebo). Although the price is the same for both indications, Tarceva^®^ clearly creates less value for pancreatic cancer patients.[110;180] Italy has an indication-specific pricing system to address this. Other EU member states and the US, struggle to implement a similar system[115;180].

Some ways to ensure that a drug is priced according to its ‘true’ value are: risk-sharing pricing, pay-for-performance pricing or outcome-based pricing. These pricing schemes allow governments and companies to renegotiate a drug’s price based on its real-life performance in terms of effectiveness, sale volumes or cost-effectiveness.[56;99;185] In this way, companies have an interest in the real-life therapy outcomes (in terms of effectiveness), and not just the drug’s performance in clinical trials (in terms of efficacy). For example, companies could offer a drug at a reduced (or no) cost and receive annuity-style payments if a drug reduces hospitalizations after approval.[2;169;180;186] Examples of outcome-based pricing exist, though only for specific indications and small populations.[169;180] Tracking the results in the real world is also a difficult administrative assignment.[187] However, this scheme has recently been implemented for several larger indications, such as for the effectiveness of a new cholesterol-lowering drug[187;188] and for the hepatitis drug Sovaldi^®^ in Japan based on volume[189].

#### 4.2.5 Setting price and profit ceilings

Another method of controlling drug pricing is to set price ceilings in various forms. For example, to combat the high prices of generic drugs in Canada, the government has recently negotiated a fixed price ceiling for six of the most used generic drugs.[190] This one-size-fits-all approach might still result in overpricing for some of the six, and be too low to supply the entire market for others. A lower price could probably be negotiated through alternative tactics, like an open-tender invitation, but the several Canadian states failed to agree on an alliance for bulk purchasing[190].

In the UK, the government signed an agreement with the pharmaceutical industry that limits increases in spending on branded medicines to below 2% per year. If more is spent, the industry has to reimburse the government.[114;119;183] Companies that did not sign this agreement are subject to direct price control[114].

One more option is to introduce upward price rigidity, by prohibiting increases in drug prices.[93;119] In the US, it is industry practice to increase the list prices of marketed drugs at least yearly by substantial amounts, synchronized with the competition.[146;191;192] Canada, however, only allows drug prices to rise with inflation.[119] This causes companies to set high initial prices, especially when the sales volume is uncertain[93].

A rather alternate approach is limiting companies’ ability to make high profits. An example of this is the Pharmaceutical Price Regulation Scheme (PPRS) in the UK.[36] If profits exceed a percentage agreed on after negotiations, a company must reduce prices, delay price increases or repay the excess to the Department of Health[36].

This approach is designed to be an incentive for pharmaceutical companies to either reduce the prices of their drugs, or invest more of their income in research. Conversely, it could also incentivize companies to spend more money on areas that are not relevant to reduce profit and avoid paying the Department of Health.

Partnerships between companies and charity foundations can help direct pharmaceutical research towards a needy cause. For example, the GlaxoSmithKline–PATH Malaria Vaccine Initiative partnership yielded Mosquirix, the first malaria vaccine.[193;194] The partnership included a payment of $200m from the PATH Malaria Vaccine Initiative, backed by the Bill and Melinda Gates Foundation, to help fund pediatric trials.[195] The price of the drug was agreed to be the costs plus 5%, which is the same price as an insecticide-treated bed net, so that it would be available for those in need.[193;194] The profit is then reinvested in more malaria research[194].

#### 4.2.6 Lowering prices through open-tender invitation and negotiations

Many countries use open tenders to lower prices.[55] They invite manufacturers or wholesalers[55] to propose a confidential price for which they can guarantee to exclusively deliver a specific drug (e.g. simvastatin) or a drug in the same class (e.g. any statin) for the entire market for a term (often two years). The supplier or suppliers who offer the lowest price receive the contract, and secure income during the contract period[99].

This approach effectively reduces drug prices, since generic drug manufacturers are stimulated to offer the lowest, but still profitable price. However, open tenders require a market size that is significant enough for companies to be interested, enough generic producers to create competition[50] and an agreement on the contract conditions[190].

A downside to this market exclusivity for generics is the risk of shortages if the winning company fails to supply.[161;190] For companies losing a bid, it could be difficult to offer a competitive price in the next round due to loss of capacity, which reduces competition and creates new monopolies[55].

Drug prices are often negotiated between national governments and companies, based on a reference price or a figure based on a drug’s cost-effectiveness and budget impact.[99] As for many schemes, increasing the number of patients for whom negotiations are held is thought to increase bargaining power and decrease prices, since companies are then less likely to deflect a low offer due to fears of losing market share.[84;190;196] New Zealand implemented a pharmaceutical management agency to have a single negotiator for the entire population, but not all countries are so organized.[78] For example, in the US, Medicare is not allowed to negotiate for drug prices[119;197]; if a drug is approved and prescribed, Medicare has to cover it, so its bargaining power is limited.[119] Other players in the highly fragmented US market are small, thus reducing everyone’s negotiating power[98].

An important downside to negotiating lower prices on a health care provider level or national level is that this might indirectly increase prices for other providers and countries, who have smaller health care budgets, which results in a weaker position to negotiate[1;145].

#### 4.2.7 Transnational licensing and pricing frameworks

To increase access to drugs that are on patent and expensive, but necessary in LMIC, these countries’ authorities can choose to issue compulsory licences as allowed by the World Health Organization (WHO).[79;198] This means that the authorities recognize the drug patents, but are allowed to have local generic manufacturers produce the same drugs, without fearing claims of patent infringement, or they can import the drug from another generic manufacturer.[7;56;79;81] This reduces the costs of a new drug dramatically,[171] though other options like international procurement seem to offer a better discount. Unfortunately, this approach is also administratively cumbersome, since in general, it applies to one drug at a time,[56] and could result in other innovators withdrawing their drug from the market.[199] However, compulsory licensing can be used successfully as part of a strategy to reduce prices offered by the originator[56;79;81;199].

International procurement is based on collective price negotiations between an innovative company and a union of LMIC. This approach leads to lower prices and more accessibility than compulsory licensing.[81] Lower prices can be achieved through voluntary out-licensing,[73] wherein the originator allows a generic manufacturer to produce the drug at reduced costs in exchange for a royalty. One example is the out-licensing of Harvoni^®^ (containing sofosbuvir and ledipasvir), which Gilead Sciences gave to an Indian manufacturer to produce for 91 LMIC, against a royalty of 7%[81].

High-income countries can also benefit from forming a union to increase bargaining power. For example, The Netherlands and Belgium recently signed an agreement to negotiate process for orphan drugs as a block. Several EU-countries have followed this example and joined the agreement, and some pharmaceutical companies have indicated their willingness to cooperate[150].

A more utopian option to regulate drug pricing is the proposal of a tiered pricing framework.[79;80] This would put a global public body, such as the WHO, in charge of regulating prices of patented drugs worldwide. It would set prices for countries based on income, disease burden and possibly the rates of out-of-pocket payments. This would differentiate prices for rich and poor countries, and achieve fairer drug prices for consumers on a global scale.

Difficulties in the execution of this plan are to obtain international consent on the rules and calculations, and prevent leakage into other markets through parallel trade. Also patent rights would have to be equally respected and be applicable for the same terms, since it would be a void policy if patent-infringing cheaper generic drugs were available.[80] Analyses of existing systems with a comparable design in LMIC have shown increased profits for pharmaceutical companies and an increased availability of medical innovations[80].

A comparable idea is that a global fund financed by governments would reward companies, based on a share of the contribution they make to global health with all their products. In exchange, those companies would have to manufacture a drug at the lowest feasible price.[1;200] Unfortunately, this approach would be extremely difficult to implement and measure.

## 5. Discussion

Taking into account the complex and interconnected dynamics of drug life cycles, pricing and intervention policies, several issues are worth mentioning and questioning. Recurring arguments in the drug pricing debate are now presented for discussion.

### 5.1. R&D through merger and acquisition

Companies are demonstrating a shift from in-house R&D to cheaper merger and acquisition-based development to fill the pipeline.[41;42;104;201] This shift is due to the reduced efficiency of in-house research,[86] and transfers the risk of failing research from big companies to small startups. In this new format, small startups go bankrupt when the research does not yield a profitable product, so that large companies don’t have to suffer the losses. In the case of a successful start-up, larger companies simply buy the licence or the entire company. In the end risk is shifted from big manufacturers to investors and governments who have supported those startups. Although this business strategy is understandable in economic terms, it provokes perverse effects in the pharmaceutical marketplace that require new forms of regulation.

Valeant,[45;114;202] Turing and Amedra [197] are examples of companies that are mostly focused on buying profitable products, instead of performing in-house R&D. Another example is Gilead Sciences, which bought sofosbuvir (Sovaldi^®^) from Pharmasset, and marketed the drug at double the cost that Pharmasset had intended to charge.[203] We seriously doubt the long-term viability of investor-centered business strategy[147;204].

Some generics are only produced by a couple of companies, so it is possible to buy all the drug rights and drastically raise prices.[16;140;184] This practice allows for a monopoly until competitors succeed in starting production as well. A major delay in this is the processing time for generic approvals, which is approximately ten months.[140] Many of the recent overnight increases in drug prices have been caused by this tactic that closely resembles a hedge fund strategy, rather than a pharmaceutical one.[16;200] This strategy was used for Daraprim^®^,[15;16] albendazole,[197] Aloquin,[20] doxycycline and thalidomide[140].

### 5.2. Patent law revision and stimulating public-private partnerships

The patent system is often blamed for high prices, because it limits competition and helps create monopolies. There certainly is an urgent need to revise the patent system in order to stimulate true innovation and prevent surrogate innovation as well as abuse of the patent system by a pharmaceutical industry that increasingly seeks to exploit the re-patenting loophole.[205] Unfortunately, removing the patent system completely would significantly reduce companies’ incentive to invest in research, so other solutions are required.[41] An alternative trajectory might be to have all drug research paid for by public money, in the same way that outcomes–prescription drugs–are currently paid for by public money. Academic institutions already perform a significant portion of new drug development, but currently lack the funds, capacity and incentives to develop a drug completely without support.[41;206] Partnerships between companies, governments, research and charity organizations, referred to as public-private financing partnerships, with agreements on the drug availability and product price, seem most promising.[193;194] This approach is what accelerated the development of the malaria vaccine, Mosquirix^®^.

New initiatives to help reduce patents’ limiting accessibility and affordability effects are currently being developed.[32] GSK announced a graduated approach, stating that it would not defend patents in the poorest countries, and transfer licences to generic manufacturers in LMIC using the World Bank classification.[207–209] This approach would increase access to new drugs without limiting profitability in high-income countries.

Public-private partnerships could also stimulate commercially unattractive but essential therapeutic innovations in high-income countries. Exemplary is the clear societal need for new antibiotics, given the increasing prevalence of multi-drug-resistant bacteria. The possible gains for the industry are too low to make innovation economically viable.[200] The population base for a new antibiotic is small, because it would be the option of last resort. In addition, the length of antibiotic treatment is short, making the volume of the market very small.[210] Extremely high prices could compensate for this situation, but it is doubtful whether society would be willing to pay. Due to low incentives in the current market, governments are tempted to impose regulation to make antibiotic development more attractive,[206;210;211] as is in place for orphan drugs. We recommend that this policy also includes strict drug pricing conditions, and that it is accompanied by antimicrobial stewardship to prevent the overuse and misuse of the new antibiotic drug.

### 5.3. Me-too drugs, R&D resources

Me-too drugs or follow-on drugs are drugs with minor chemical variations relative to a drug already on the market within a given therapeutic class.[173] These drugs are highly controversial since they often cost roughly the same as the first-in-class drugs, but offer few relevant therapeutic improvements.[68;69] Me-too drugs are seen as an ineffective consumption of R&D resources and diminishing incentives for innovation[24].

Another side of the argument is that me-too drugs increase choice, and make treatment available for certain groups, or help match a drug’s pharmacokinetics, effectiveness or side effects in specific populations.[24;68] Me-too drugs are also a consequence of R&D races between multiple companies developing drugs for the same indication, so they are inherent to a competitive system.[173;212] Given that a first-in-class drug has a significant advantage in market share and costs, this competition forces companies to speed up innovation[69].

Studies suggest that the price of me-too drugs only falls after the third introduction.[93;173] Where the first movers compete over quality only, the drugs introduced later compete over price to overcome the disadvantage of being a new drug with no clinical record.[212] This holds for Gilead Sciences’ hepatitis C drugs as well, where MSD is competing with Zepatier^®^ with a price tag that is 40% lower than the first-in-class[115;213].

### 5.4. Lack of systematic research into policy effectiveness

Various policies related to drug prices with roughly the same aim have been introduced around the world, but comparing the effectiveness across policies appears difficult.[41] Policies often lack a scientific basis, and an evaluation after the policy is introduced is not always performed, let alone in a standardized way.[41;84;165] Insights on the long-term policy effects of for instance value-based (QALY-guided) pricing are particularly scarce,[165] which causes some countries to pay unnecessarily high prices for medicines.[35;163] Even regulatory guidelines are implemented without evaluation,[41] which causes some to ask for lowered regulation to speed up innovation[66] and others to ask for more regulation for additional safety. Unfortunately, both groups lack the evidence to back their case. Given the current trends in big data analytics, better monitoring of the effects of drugs with respect to health outcomes and the effects of policy on pricing should be possible.[89] Health Technology Assessment methodologies (HTA) could be used to strengthen evaluation of policy measures. Furthermore, the option of involving regulatory gatekeepers of safety and effectiveness like the FDA and the EMA in drug pricing policies should be considered more seriously. Like drugs, drug policy must be evidence-based[110].

### 5.5. Justification of drug prices

Life expectancy has gone up by 30 years in just a century in high-income countries.[77] It can be stated that innovation is always expensive, as seen in other new technologies, and once the price of innovation has been paid, generics make innovative treatments widely available. Revolutions in biotechnology, nanotechnology, biophysics and genome sequencing have moved precision medicine from the bench to the bedside,[53;89;214] yielding a burst of innovation in medicine.[30;42;75;160] This helps to explain the large number of new and expensive targeted therapies that have come to the market recently. Cancer mortality has been reduced by 20% in the last two decades[77] through the introduction of new drugs, but that is also due to screening, prevention, vaccination and surgical improvements[58].

Steep pricing strategies are historically an oncological phenomenon,[91;214] and recently prices are rising to even higher levels.[94] This is peculiar, since some oncological drugs offer only small benefits.[5] In addition to this low effect, oncological drugs are frequently priced so high that they are not cost-effective.[215] What makes the situation more financially challenging is that cancer drugs are often more effective in combinations,[75] thus making therapy cost the sum of the drugs.[98] Debates about this situation have usually ended in the consensus that patients cannot be denied new cancer treatments simply because of costs,[214] so eventually most cancer drugs have been reimbursed despite the high costs. But this is no longer sustainable given the steadily growing patient populations with cancer and stagnating health budgets.

As more treatments become possible, expectations rise.[58] Diseases that were a death sentence decades ago are now treatable, and leaving them untreated is not acceptable. With increasing possibilities come increased demands and increasing costs[216].

These innovations have allowed for more personalized diagnosis and cures, leading to precision or personal medicine, aimed at highly stratified patient populations. This gives better outcomes, but given the often smaller populations that qualify for a treatment (and thus smaller volumes of sales) prices are high to generate adequate revenue.[214] This shift from blockbuster to niche buster will make many untreatable diseases treatable, but costs for the healthcare sector will continue to rise[217].

### 5.6. Patient-assistance programs and list prices

Companies that raise prices often defend their actions by stating that patients who cannot afford the drugs are offered assistance in the form of patient assistance programs programmes in Western countries.[156] These programs allow patients to apply for the drugs at reduced or no cost, if they are uninsured and live below a certain income level.[114] The income level is set so that many patients on normal wages don’t qualify, so that drug prices can result in catastrophic spending.[114] Furthermore, patient-assistance programs increase the workload for general practitioners’ assistants, since they often require many forms.[140] The costs to the healthcare system are still unnecessarily large, and are shifted from patients to insurance companies.[141;154;218] These programs basically allow companies to sponsor the purchase of their own drugs[147].

Another argument for defending high prices is that pharmaceutical companies do not actually charge list prices for drugs. Hospitals and insurers often negotiate discounts on drug list prices,[142;156;219] and sometimes up to 50%.[141] However, drug prices worldwide remain at unrealistically high levels, since examples of annually raising prices (or prices rising after selling licences) to more than double the original price are plentiful[114;220].

In some cases, companies provide free drugs for a small market simply because they feel they are morally obliged to do so. These are usually small companies who do not want to invest in regulatory approval procedures.[70] Larger companies see it as a moral obligation to maximize sales volumes and profits, reach patients and secure future investments in research, continue innovation,[94] and pay their stock holders.

Trying to defend high spending on pharmaceuticals, companies often point out that in the US only $300 billion is spent annually on prescription medicines compared to the $1 trillion that is spent on hospital care.[2;184] Therefore, compared to total healthcare spending, US pharmaceutical costs are just 10% of the total health care budget, which is roughly the same throughout Europe.[184] This number has remained constant for over 50 years.[77;221] Thus, all other healthcare costs, such as hospitals and general practitioners’ costs, have risen at the same rate as pharmaceutical costs.[39] It is simply easier for governments to reduce drug budgets than to reduce hospital staff wages or restructure inefficient healthcare infrastructures.[2] However, this still does not justify high drug prices. Just because other parts of the system are not functioning as efficiently as possible, does not mean pharmaceutical companies should be increasing prices at the same rate.

### 5.7 Methodological notes

This paper used both scientific publications and newspaper articles from several selected newspapers. The selection of those sources allowed for a comprehensive view on the subject of drug pricing. Clear differences in focus between the newspaper articles and scientific publications were observed.

The newspaper articles tended to focus on public opinion in one country based on one event, with examples and personal stories about the impact of high drug prices on patients’ lives. A thorough analysis on causes and policies of drug pricing was often missing, and a rather mono-dimensional conclusion was frequently offered that pharmaceutical companies choose to keep the prices high for their own exorbitant profits.

The journal articles offered more policy-focussed analyses of the causes of and solutions to high drug pricing. They more frequently covered in-depth discussions of single policy measures per country and their proposed or measured effect, but often left the patient perspective out.

In conclusion, the combination of both peer-reviewed scientific papers and newspaper articles allowed for a significantly contextualized evidence based conclusion. The mixed-method approach yielded deeper insights into the problem area of high drug pricing and sustainable drug markets than one of the two individual methods would have been able to achieve alone.

## 6. Conclusion

The current rise in drug prices worldwide is making healthcare unaffordable even in high-income countries. Apart from historic changes in the drug life cycle dynamics, price-volume proportions, and a transition from “one-size-fits-all” to more stratified precision medicine approaches, this problem is due to patent-induced monopoly positions, unintended consequences of drug and reimbursement policies and competitive market failure. This situation threatens to disturb the fragile compromise between the basic human right for affordable access to healthcare and the utilitarian protection of inventions to incentivize innovation. The current pricing spiral will only stop through well-designed regulatory interventions and measures around drug pricing on a national and transnational levels. Access to medicines needs to be central to any policy intervention discussion—something that can be overlooked when governments are arguing for lower health care costs to reduce spending, while the pharmaceutical industry is repeating the argument for competitive pricing to reimburse their R&D costs.

Many options to regulate the pharmaceutical market have been tried, some with better success than others. It is clear that reference pricing–both internal and external–and incentivizing physicians and pharmacists are the fastest, most effective and most reviewed options. Transnational cooperation, for example through the European Union, African Union, World Bank or WHO, would help reduce drug prices with increased bargaining power, and could potentially reduce administrative costs. Apart from creating collective negotiating power transnational cooperation would also stimulate exchanging trial data, sharing patient records and improving evaluation methods. A global framework for cooperation among drug regulatory authorities (e.g. FDA and EMA) would increase those benefits even more, and could be further amplified by reinforcing the existing WHO framework that already helps to reduce drug prices by means of an essential medicines list, which facilitates compulsory licensing.

Reduced healthcare spending is thought to reduce incentives for innovation, but given the current double-digit profit margins, industrial incomes could be lower without harming the industry’s outlooks. Public-private partnerships, in which charity funds are used to sponsor research in exchange for lower prices, could significantly help direct spending decisions on research away from primarily financial motives towards what is best for society.

Value-based pricing is a promising but also risky option that is already being used by some countries to reduce costs. The (inter-)national public debates about how much a QALY should cost and the regulatory and policy debates about whether and how to continue with the QALY appraisal tools still have to reach a conclusion. Until consensus is reached, drug companies will continue to strategically use the QALY and/or ICER thresholds to boost their profits. Governments should take this into account while continuing to deploy sophisticated ICER-based compensation models in the era of precision medicine.

In conclusion, the recent rise in drug prices is caused by uncontrolled market dynamics, changes in life-cycle dynamics and unanticipated policy side-effects. There is a wide range of policy tools to reduce drug prices available through various mechanisms. The most effective options are reference pricing and incentivizing physicians and pharmacists, whereas for the long term value-based and outcome-based pricing next to public-private partnerships are most promising developments. The challenge is, of course, how to strike a balance between rewarding investments in innovation, achieving reasonable drug pricing for governments and securing equitable access to medicines.

## Supporting information

S1 PRISMA Checklist PRISMA 2009 checklist—Jun2017.docx. (DOCX)
